# Antimicrobial Effect of the Triterpene 3***β***,6***β***,16***β***-Trihydroxylup-20(29)-ene on Planktonic Cells and Biofilms from Gram Positive and Gram Negative Bacteria

**DOI:** 10.1155/2014/729358

**Published:** 2014-06-29

**Authors:** Francisco Flávio Vasconcelos Evaristo, Maria Rose Jane R. Albuquerque, Hélcio Silva dos Santos, Paulo Nogueira Bandeira, Fábio do Nascimento Ávila, Bruno Rocha da Silva, Ariana Azevedo Vasconcelos, Érica de Menezes Rabelo, Luiz Gonzaga Nascimento-Neto, Francisco Vassiliepe Sousa Arruda, Mayron Alves Vasconcelos, Victor Alves Carneiro, Benildo Sousa Cavada, Edson Holanda Teixeira

**Affiliations:** ^1^Integrated Laboratory of Biomolecules (LIBS/BioMol-Group), Department of Pathology and Legal Medicine, Faculty of Medicine, Federal University of Ceará, Fortaleza 60430-160, CE, Brazil; ^2^Centre of Exact Sciences and Technology, Acaraú Valley State University, 62040-370 Sobral, CE, Brazil; ^3^Biologically Active Molecules Laboratory (BioMol-Lab/BioMol-Group), Department of Biochemistry and Molecular Biology, Federal University of Ceará, Fortaleza 60440-970, CE, Brazil

## Abstract

This study evaluated the antimicrobial effect of 3*β*,6*β*,16*β*-trihydroxylup-20(29)-ene (CLF1), a triterpene isolated from *Combretum leprosum* Mart., in inhibiting the planktonic growth and biofilms of Gram positive bacteria *Streptococcus mutans* and *S. mitis*. The antimicrobial activity was assessed by determining the minimum inhibitory concentration (MIC) and minimum bactericidal concentration (MBC). The antibiofilm potential was determined by quantifying total biomass and enumerating biofilm-entrapped viable bacteria. In addition, the acute toxicity of CLF1 on *Artemia* sp. nauplii was also determined. The results showed that CLF1 was able in inhibiting the growth of *S. mutans* and *S. mitis* with MIC and MBC of 7.8 *μ*g/mL and 15.6 *μ*g/mL, respectively. CLF1 was highly effective on biofilms of both bacteria. Only 7.8 *μ*g/mL CLF1 was enough to inhibit by 97% and 90% biomass production of *S. mutans* and *S. mitis*, respectively. On the other hand, such effects were not evident on Gram negative *Pseudomonas aeruginosa* and *Klebsiella oxytoca*. The toxicity tests showed that the LC_50_ of CLF1 was 98.19 *μ*g/mL. Therefore, CLF1 isolated from *C. leprosum* may constitute an important natural agent for the development of new therapies for caries and other infectious diseases caused by *S. mutans* and *S. mitis*.

## 1. Introduction

The resistance to available antimicrobials is currently a public health concern in the world. Several types of infectious diseases have become difficult to treat and expensive to cure mainly due to the low efficiency of antimicrobials for current bacteria [1, 2]. Moreover, the socioeconomic impact caused by infectious diseases is quite significant as shown by the high amount of financial resources spent on the clinical treatment of patients [3, 4]. Taking this into consideration, the ability of microorganisms to grow as biofilms and the increased rate of microbial resistance to conventional antibiotics contribute to the expanding epidemiology of infectious diseases [5].

Biofilms are complex microbial communities that establish themselves on a wide variety of surfaces and are generally associated with an extracellular matrix consisting of different polymers [6, 7]. Biofilms provide protection against the action of antibiotics and supplies a barrier to prevent or reduce the penetration of antimicrobial agents through the matrix [8]. The eradication of microorganisms that live as biofilms becomes extremely difficult or even impossible [9, 10]. Therefore, the search for new molecules able to inhibit biofilm formation, either by inhibiting microbial growth or by inhibiting their adhesion to a substrate, has intensified in recent years.

The shrub* Combretum leprosum *Mart. is popularly known in Brazil as “mufumbo” or “cipoaba” and is commonly found in the northeast. In folk medicine, it is widely used as an expectorant, antimicrobial, and antihemorrhagic agent [11].

Although scarce, research on the biological activities of* C. leprosum* points to similar actions to those observed in other species of the same genus. Nunes and coworkers [11] provided evidences about gastroprotective and antiulcerogenic roles of the ethanolic extract (EE) from the bark of* C. leprosum*, which were attributed to the increase in mucus production and inhibition of gastric acid secretion in rats.

In the present study, we evaluated the antimicrobial potential of the triterpene 3*β*,6*β*,16*β*-trihydroxylup20(29)-ene (CLF1) isolated from leaves of* C. leprosum* Mart. on the Gram positive bacteria* Streptococcus mutans* and* S. mitis* and on the Gram negative bacteria* Pseudomonas aeruginosa* and* Klebsiella oxytoca*.

## 2. Materials and Methods

### 2.1. Strains and Culture Conditions

The Gram positive strains* Streptococcus mutans* UA159 (ATCC 700610) and* S. mitis* (ATCC 903) and the Gram negative strains* Pseudomonas aeruginosa* (ATCC 10145) and* Klebsiella oxytoca* (ATCC 13182) were kindly provided by the Oswaldo Cruz Institute (FIOCRUZ, Rio de Janeiro, Brazil). The growth of all strains started from a stock culture maintained at −80°C in BHI broth (Brain Heart Infusion, Himedia, Mumbai, India) with 20% glycerol. Each microorganism was inoculated in 10 mL of fresh sterile BHI broth and incubated for 24 hours at 37°C with or without 10% CO_2_ for Gram positive and Gram negative, respectively. After the initial activation, the culture was renewed by transferring 100 *μ*L of inoculum into 10 mL of new sterile BHI broth and grown under the same conditions as previously reported.

### 2.2. Plant Material

Fresh leaves of* C. leprosum* were collected in June 2009 in Salgado dos Machados district, located 15 km from the city of Sobral, Ceará, Brazil. The plant authentication was performed by Professor Elnatan Bezerra de Souza, a plant taxonomist from Acaraú Valley State University (Sobral, Brazil), and a voucher specimen (N° 4573) has been deposited at the herbarium Francisco José de Abreu Matos (Sobral, Brazil).

### 2.3. Sample Preparation

Fresh leaves (2.5 kg) of* C*.* leprosum* were powdered and extracted at room temperature with an EtOH/H_2_O solution (8 : 2 v/v). After 15 days, the resulting material was subjected to a simple filtration, followed by evaporation of the solvents under reduced pressure. An aliquot of the obtained solution was then lyophilized, resulting in a crude ethanol extract (EECL). The EECL was suspended in MeOH/H_2_O 3 : 1 and partitioned with CH_2_Cl_2_, EtOAc, and* n*-BuOH. The CH_2_Cl_2_ extract was evaporated under reduced pressure, yielding a residue (15.5 g) that was fractioned over a Si gel and eluted with hexane/EtOAc (8 : 2, 6 : 4, 2 : 8), EtOAc, and MeOH. The hexane/EtOAc (6 : 4) fraction (4.19 g) was chromatographed over a Si gel and eluted with hexane/EtOAc (in a gradient of 9.5 : 0.5 to 0.5 : 9.5), EtOAc, and MeOH, resulting in 130 fractions which, after thin layer chromatography (TLC), were combined into 10 fractions (F_1_–F_10_). The F7 fraction (528 mg, resulting from elution with hexane/AcOEt 6 : 4) was rechromatographed using the same solvent system to purify CLF1 (54 mg).

### 2.4. Structural Analysis by Infrared Spectroscopy and Nuclear Magnetic Resonance

IR spectra were recorded using a Perkin-Elmer 1000 spectrophotometer. ^1^H and ^13^C NMR were recorded on a Bruker Avance DPX-500 (500 MHz for ^1^H and 125 MHz for ^13^C); chemical shifts are given in ppm (*δ*_C_ and *δ*_H_) relative to CDCl_3_ (7.27) and (77.23).

### 2.5. Sample Preparation for Antimicrobial and Toxicity Assays

CLF1 was first solubilized in 99.9% dimethyl sulfoxide (DMSO), and then the concentration was adjusted to 62.5 *μ*g/mL in sterile distilled water to a final concentration of 8% DMSO and serially diluted to obtain concentrations ranging from 31.25 to 3.9 *μ*g/mL. The ethanolic extract of* C*.* leprosum* (EECL) was equally evaluated regarding its antimicrobial potential. Briefly, EECL was first solubilized in 99.9% DMSO, and then the concentration was adjusted to 500 *μ*g/mL in sterile distilled water to a final concentration of 5% DMSO and submitted to twofold serial dilutions to obtain concentrations ranging from 250 to 31.25 *μ*g/mL. Chlorhexidine at 125 and 31.25 *μ*g/mL and distilled water with 8% DMSO were used as positive and negative controls, respectively.

### 2.6. Antibacterial Activity

The antibacterial effects of CLF1 and EECL were evaluated through a microdilution test in 96-well “round-bottom” polystyrene plates. First, the wells were filled with 100 *μ*L of the bacterial suspension at 2 × 10^7^ CFU/mL. Then, 100 *μ*L of CLF1 or EECL was added at different concentrations as previously described, obtaining a final volume of 200 *μ*L per well. The plates were incubated for 24 hours at 37°C with or without 10% CO_2_ for Gram positive and Gram negative, respectively.

The minimum inhibitory concentration (MIC) for each microorganism was determined to be the lowest CLF1 or EECL concentration that showed a complete inhibition of visible bacterial growth. To determine the minimum bactericidal concentration (MBC), 10 *μ*L from each well, in which no visible growth was detected, was inoculated in Petri dishes containing fresh BHI agar and incubated at 37°C for 24 hours. MBC was considered to be the lowest concentration of compounds able to completely inhibit microbial growth on the plates.

### 2.7. Antibiofilm Activity

#### 2.7.1. Biomass Quantification

Sterile 96-well “flat-bottom” plates were prepared using a procedure similar to that used in the antimicrobial activity tests, with the same concentration of cells. All plates were incubated on a horizontal shaker (120 rpm/min) at 37°C for 24 hours for biofilm development in the presence of CLF1 or EECL. Then, the supernatant of each well was removed and biofilms washed three times with 200 *μ*L/well sterile water to remove cells weakly adhered. The attached biofilm mass was quantified using crystal violet (CV) staining [12]. Briefly, the plates containing biofilms were air dried for 30 minutes, and 200 *μ*L of 99% methanol was transferred to each well and incubated for 15 minutes to fix the adhered cells. Then, the methanol was removed followed by addition of 200 *μ*L/well of 0.1% CV (Gram-staining set for microscopy; Merck) and the plates were incubated for 5 minutes. After the staining step, the washing process was repeated with sterile water, and the plates were left to dry at room temperature. In order to solubilize the dye bounded to the biomass, 200 *μ*L of 33% (v/v) acetic acid (Merck) was added to each well and the plates were agitated for 15 minutes on a horizontal shaker. The CV solutions were transferred to a new 96-well plate and the optical density of the content was measured using a plate reader (BioTrak II-Amersham Biosciences) at 590 nm.

#### 2.7.2. Biofilm Cell Enumeration

In order to determine the effect of CLF1 on the viability of biofilm-entrapped cells, biofilms' suspension was prepared according the same conditions as previously described. After 24 hours of incubation at 37°C, the supernatant was discarded and the plates were washed with sterile water to remove the weakly adhered cells. Then, 200 *μ*L of sterile water was added to each well and the plates were subjected to ultrasonic bath for 15 minutes to remove the biofilm-entrapped cells. The volume corresponding to five wells of each condition was then collected and pooled in a sterile 1.5 mL tube. The suspensions were serially diluted, plated on BHI agar, and incubated at 37°C for 24 hours. The number of colony-forming units (CFU) was determined and expressed as CFU/mL.

### 2.8. Acute Toxicity on* Artemia *sp

#### 2.8.1. Nauplii Harvesting

To obtain viable nauplii, 30 mg of cysts was weighed and hydrated for 1 hour in distilled water under constant aeration. After hydration, the cysts were treated with 50% sodium hypochlorite under constant agitation, until the color of the cysts changed from brown to orange. In addition to acting in the corium layer by facilitating the nauplii outbreak, sodium hypochlorite also promotes cleansing of the cysts. When the cysts reached the orange color, they were immediately washed with distilled water to remove the sodium hypochlorite. The cysts were then transferred to a conical bottom tube containing filtered seawater under constant aeration and after 48 hours, the nauplii were ready to use in toxicity tests [13].

#### 2.8.2. Toxicity Test

CLF1 was solubilized in seawater plus DMSO 8% to obtain a stock solution of 125 *μ*g/mL. All tests were performed using 5 different concentrations of CLF1 (125, 62.5, 31.25, 15.6, and 7.8 *μ*g/mL). A group with seawater with 8% DMSO was used to verify whether DMSO was toxic to the nauplii. Seawater alone was used as the negative control. All tests were carried out in triplicate and repeated three times independently. The assay was performed in 24-well polystyrene plates with 10 nauplii per well and the mortality was determined after 24 and 48 hours by analysis of the number of nauplii devoid of motility. To determine the LC_50_, the data were calculated and analyzed using the TSK (Trimmed Spearman-Karber) statistical program. Up to 10% mortality was considered acceptable in the negative control [13].

### 2.9. Statistical Analysis

Statistical analysis was performed by GraphPad Prism version 3.00 for Microsoft Windows. The method used was one-way ANOVA with Bonferroni* post hoc* test. The data were recorded in triplicate from at least three separate experiments and graphs are presented as mean ± standard deviation. The data were considered significant when *P* < 0.001.

## 3. Results

### 3.1. Purification and Structural Analysis of CLF1

**Figure 1:**
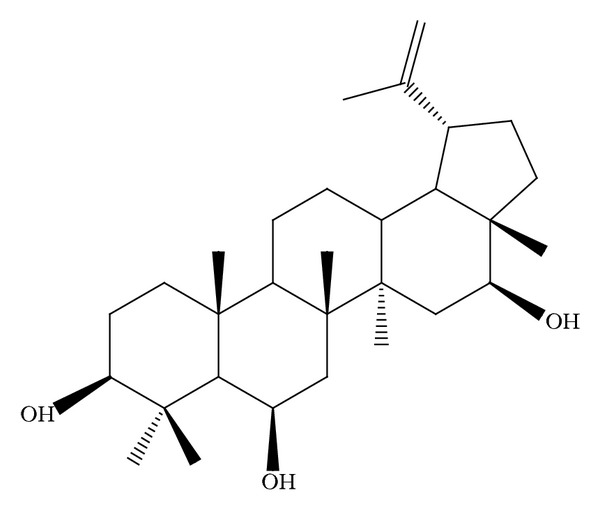
Structure of 3*β*,6*β*,16*β*-trihydroxylup-20(29)-ene extracted from* Combretum leprosum*.

CLF1 was purified as a white solid. Its infrared spectrum (IR) showed a broadband that indicated the presence of hydroxyl groups (3463 cm^−1^), as well as absorptions at 1643 and 883 cm^−1^ corresponding to a 1,1-disubstituted double bond moiety. This moiety was confirmed by chemical shifts of ^13^C [*δ*_C_ 150.1 (C-20) and 110 (C-29)] and ^1^H [*δ*_H_ 4.75 (br s, H-29a) and *δ*_H_ 4.64 (br s, H-29b) NMR. The presence of three hydroxyl groups were supported by a chemical shift at *δ*_C_ 76.9, 69.0, and 79.3, which, in the HSQC spectrum, showed correlations with the hydrogens at *δ* 4.52 (br s; H-16), 3.58 (dd,* J* = 11.2 and 4.4; H-6), and 3.12 (t,* J* = 6.7; H-3, resp.). Comparison of the chemical shifts with the literature data [14] revealed that CLF1 is the triterpene of the lupane skeleton named 3*β*,6*β*,16*β*- trihydroxylup-20(29)-ene, which was previously isolated by Facundo and coworkers [15] (Figure 1).

### 3.2. MIC and MBC

Both EECL and CLF1 inhibited the growth of* Streptococcus mutans* and* S. mitis*. However, CLF1 presented lower MIC and MBC values when compared to EECL (Table 1). On the other hand, the same effect was not seen on Gram negative* Pseudomonas aeruginosa* and* Klebsiella oxytoca* (data not shown).

When compared to EECL, CLF1 inhibited the growth of* Streptococcus mutans* more than 16-fold (Table 1).

### 3.3. Antibiofilm Activity

**Figure 2:**
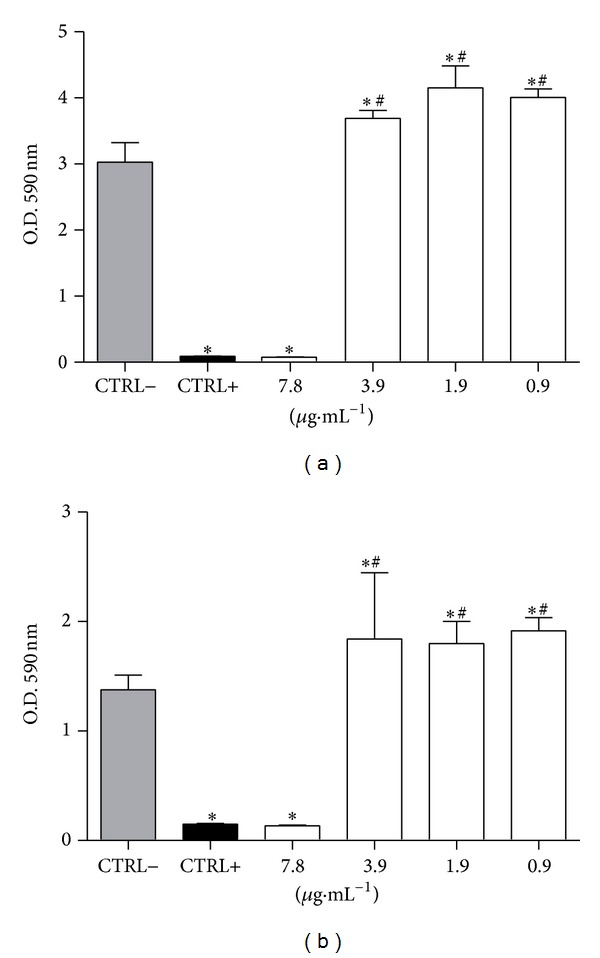
Effects of CLF1 on (a)* Streptococcus mutans* and (b)* S. mitis* biofilm mass. Biofilms were grown for 24 hours in the presence of CLF1 at different concentrations. The negative control was performed with 8% DMSO and the positive control with chlorhexidine at 31.25 *µ*g/mL, and both are diluted in ultrapure water. Error bars represent standard deviation and statistical *P* value (represented by ∗ or #) indicates concentrations that are significantly different from negative and positive controls, respectively. *P < 0.001; ^#^ *P* < 0.001.

Regarding antibiofilm activity, the results showed that CLF1 was effective only on biofilms of Gram positive strains. At a concentration of 7.8 *μ*g/mL, CLF1 inhibited the biomass of* S. mutans* and* S. mitis* by 97% and 90%, respectively. For EECL at 125 *μ*g/mL the inhibition was approximately 97.3% for* S. mutans* and 44% for* S. mitis*. According to these findings, the CLF1 efficiency at 7.8 *μ*g/mL is quite similar to that of chlorhexidine at 31.25 *μ*g/mL for both bacteria (Figure 2).

**Figure 3:**
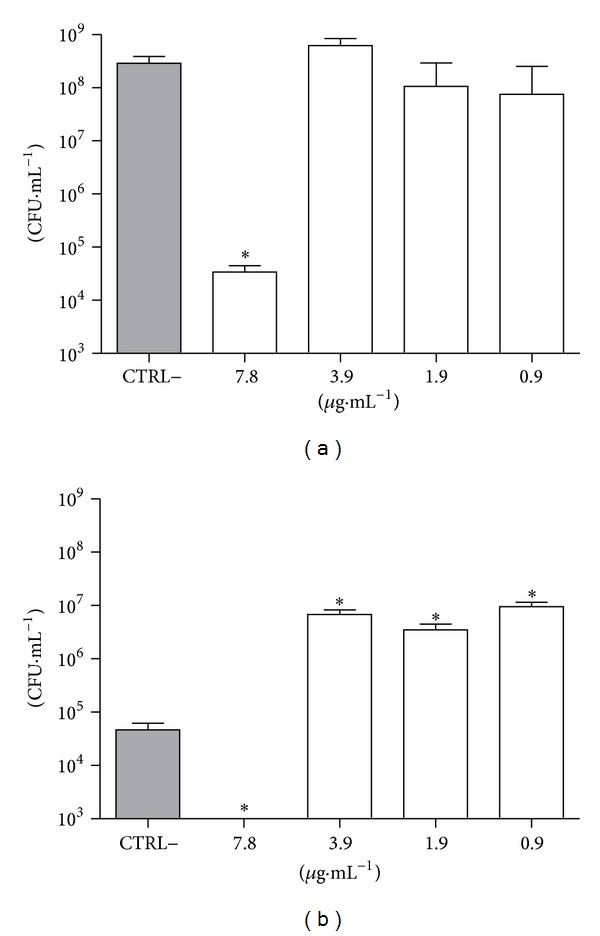
Effect of CLF1 on viability of biofilm-entrapped cells from (a)* Streptococcus mutans* and (b)* S. mitis*. Biofilms were grown for 24 hours in the presence of CLF1 at different concentrations. The negative control was performed with 8% DMSO and the positive control with chlorhexidine at 31.25 *µ*g/mL, and both are diluted in ultrapure water. Error bars represent standard deviation and statistical *P* value (represented by ∗ or #) indicates concentrations that are significantly different from negative and positive controls, respectively. *P < 0.001; ^#^ *p* < 0.001.

In order to evaluate the effect of CLF1 on biofilm-entrapped cells,* S. mutans* and* S. mitis* biofilms were grown in the presence of CLF1 at concentrations ranging from 7.8 to 0.95 *μ*g/mL. The results showed that CLF1 at 7.8 *μ*g/mL significantly decreased the viability of bacterial cells (Figure 3).

### 3.4. Toxicity Analysis

Analysis of acute toxicity with* Artemia* nauplii was carried out using 5 different concentrations of CLF1. In order to determine the LC_50_, the number of* nauplii* deprived of motility was evaluated after 24 hours of coincubation. The results showed that CLF1 presented a LC_50_ of 98.19 *μ*g/mL, which is significantly higher than the concentration that kills bacteria in antimicrobial assays.

## 4. Discussion

The search for new molecules with remarkable antimicrobial activities has become an important area of study. Moreover, natural products are a rich source for discovery of new antimicrobial agents [16]. According to Newman and Cragg [17], studies involving medicinal plants with antimicrobial activities have great potential mainly due to the necessity of plants to produce natural products that protect them against infections.

Pathogenic microorganisms that form biofilms are the focus of intense research due to their involvement in a large number of chronic infections and their role in the colonization of medical instruments, such as cardiovascular devices [18]. These findings indicate an increasing need for more effective antimicrobial agents with lower costs [19].

Studies about the phytochemical and biological characteristics of* C*.* leprosum* can be readily found in the literature. Among them, a study by Facundo and coworkers in 1993 established the structures of three triterpenoids belonging to the lupane series [3*β*,6*β*,16*β*-trihydroxylup-20(29)-ene], oleanane (arjunolic acid) and cycloartenol (mollic acid), and two flavonoids derived from the quercetin (3-*O*-methylquercetin and 3-*O*-*α*-L-rhamnopyranosylquercetin) [15].

Triterpenes belong to class of terpenoids, which are chemically characterized by the presence of six isoprene units with a total of 30 carbon atoms. Such molecules are responsible for different biological activities found in plant extracts and essential oils [20].

The bioactive metabolite 3*β*,6*β*,16*β*-trihydroxylup-20(29)-ene (CLF1) was isolated from the dichloromethane fraction of ethanolic extract of* C. leprosum* leaves (EECL). This compound appears as a white solid with an average yield of 0.02%. As previously reported by Facundo and coworkers [15], this molecule was first isolated from the hexane and ethanolic extracts of leaves and roots of* C. leprosum* and identified as a triterpene of the lupane class, with a double bond between carbons 20 and 29.

Recently, Horinouchi and colleagues [21] provided important evidences about the antiproliferative and anti-inflammatory role of the ethanolic extract of flowers from* C. leprosum*. According to the authors, the extract must be considered as a new potential tool for the treatment of several skin inflammatory diseases, since it reversed the skin inflammatory and hyperproliferative process in a very significant manner.

In the present work, the antimicrobial activity of CLF1 was assessed on Gram positive bacteria* Streptococcus mutans* and* S. mitis*. MIC and MBC values for both bacteria are 7.8 and 15.6 *μ*g/mL, respectively. No effects were seen on the growth of* Pseudomonas aeruginosa* and* Klebsiella oxytoca*, both Gram negative bacteria. Such MIC value is lower than that found by a casbane diterpene isolated from barks of* Croton nepetaefolius*, which wasevaluated on oral streptococci, including* S. mutans *and* S. mitis* [22]. Furthermore, several triterpenes isolated from different species from the genus* Miconia* presented antibacterial activity against oral streptococci with MIC ranging from 30 to 200 *μ*g/mL [23].

*S. mutans* is a Gram positive bacterium highly acidogenic and aciduric, and several clinical and laboratory studies point to the involvement of this species as the primary pathogen in human dental caries [24]. Moreover, according to Gross and coworkers [25],* S. mutans* was the most abundant species observed in samples from children with caries.

Some studies have shown that Gram positive bacteria are more susceptible to the action of plant extracts that contain flavonoids and triterpenes [26]. Regassa and Araya [27] tested different species of the genus* Combretum *and elucidated the antimicrobial potential of* Combretum molle* ethanolic extract against* Staphylococcus aureus* and* S. agalactiae*, both Gram positive bacteria.

The effect of CLF1 on biofilm development was similar to that found on planktonic growth. CLF1 at 7.8 *μ*g/mL inhibited biofilms' mass by 97% and 90% for* S. mutans* and* S. mitis*, respectively, and reduced the number of biofilm-entrapped cells. The effect of CLF1 on the inhibition of biofilm formation is likely associated with a decrease in cellular viability since the concentrations inhibiting the growth of biofilms and planktonic cells are similar. Sá and coworkers [22] found a diterpene with inhibitory properties on* Streptococcus mutans* biofilm-entrapped cells. Such diterpene inhibited* S. mutans* biofilms in 94.28%. In addition, Raja and coworkers [28] showed that a pentacyclic triterpenoid (acetyl-11-keto-b-boswellic acid) inhibited the biofilm formation and reduced preformed biofilms from* S. mutans* and* Actinomyces viscosus* at concentrations ranging from 16 to 128 *μ*g/mL. Nevertheless, other chemical structures based on terpenoids have been described in the literature as molecules possessing antimicrobial activities [29–32].

Studies from 1987 to 2004 involving secondary metabolites suggest that the antibacterial activity exerted by these compounds may be associated with mechanisms such as damage to the plasma membrane (perforation and/or reduction in membrane fluidity) [33], inhibition of nucleic acid synthesis (inhibition of topoisomerases), and inhibition of the energetic metabolism (caused by inhibition of reductase and NADH cytochrome-c) [34].

Katerere and coworkers [35] reported that some pentacyclic triterpenes isolated from* Combretum* and* Terminalia* genera proved to be ineffective against Gram negative bacteria. In such study, CLF1 showed no significant effects on the growth of* Escherichia coli*.

Thus, based on the mechanisms of action of the compounds cited above, and considering the results of our study, the antimicrobial activity of CLF1 may be related to its hydrophobicity. This feature probably allows a nonspecific interaction with cell membrane phospholipids of Gram positive bacteria. The presence of an outer membrane composed by phospholipids, lipoproteins, and, particularly, lipopolysaccharides (LPS) in Gram negative probably blocks the interaction of the molecule with the plasma membrane [36, 37].

Finally, a limiting factor in the prospection of new molecules with antimicrobial potential is the toxicity exhibited by several compounds. In the present study, the LC_50_ of CLF1 on* Artemia* nauplii is higher than its MIC. Thus, the results are very promising and CLF1 must be considered as a new antimicrobial agent against infections by* S. mutans* and* S. mitis*, including their associated biofilms. On the other hand, CLF1 is not indicated for infections caused by Gram negative bacteria, since only a small interference was seen on* P. aeruginosa* growth.

## Figures and Tables

**Table 1:** Values of MIC, MBC, and biomass inhibition of *Streptococcus mutans* and *S. mitis* treated with ethanolic extract (EECL) and 3*β*,6*β*,16*β*-trihydroxylup-20(29)-ene (CLF1) from *Combretum leprosum*.

Microorganism	MIC (*μ*g/mL)		MBC (*μ*g/mL)		Biomass inhibition (%)	
	EECL	CLF1	EECL	CLF1	EECL	CLF1
*Streptococcus mutans *	125	7.8	250	15.6	97.3	97
*Streptococcus mitis *	62.5	7.8	125	15.6	44	90
